# Morphology of the Patellar Tendon and the Contractility Response of the Quadriceps: Symmetry and Gender Analysis

**DOI:** 10.3390/ijerph18105309

**Published:** 2021-05-17

**Authors:** Pablo Abián, Fernando Martínez, Fernando Jiménez, Javier Abián-Vicén

**Affiliations:** 1 Faculty of Humanities and Social Sciences, Comillas Pontifical University, 28049 Madrid, Spain; pabloo9@hotmail.com; 2 Performance and Sport Rehabilitation Laboratory, Faculty of Sport Sciences, University of Castilla-La Mancha, 45071 Toledo, Spain; fermasa83@gmail.com (F.M.); josefernando.jimenez@uclm.es (F.J.)

**Keywords:** tendon morphology, contractility response, isometric strength, isokinetic strength, quadriceps

## Abstract

The purpose of the study was to describe the differences between the dominant and non-dominant leg regarding contractility response and quadriceps strength and the morphology and stiffness of the patellar tendon (PT) in a group of physically active men and women. Fifty physically active subjects (36 men and 14 women) were evaluated for morphology and stiffness of the PT, contractility response of the rectus femoris of the quadriceps, isometric strength of the quadriceps and hamstrings, and isokinetic strength (concentric and eccentric) at 60°/s of the knee extensors. The measurements were made on the subject’s dominant and non-dominant leg. The men showed a greater thickness of the PT in both legs compared to the women. Regarding the contractility response, the women recorded a 10.1 ± 16.2% (*p* = 0.038) greater contraction time (ct) in the dominant versus the non-dominant leg and the men recorded 11.9% (*p* = 0.040) higher values in the dominant leg compared to the women. In all the absolute strength measurements the men recorded higher values (*p* < 0.05) than the women, however, when the values were normalised with respect to the muscle mass of the leg these differences disappeared. The dominant leg showed values of isometric strength and eccentric strength at 60°/s (*p* < 0.05) greater than the non-dominant leg. The reference values provided in this study of the knee extensors and PT may be useful for detecting possible muscular or tendinous anomalies.

## 1. Introduction

The force generated by muscles is responsible for the movement of the human body, the stability of a determined joint and maintenance of body posture [1]. The capacity of a muscle to contract is an indispensable requisite during physical activities [2]. The assessment of human strength is used to evaluate physical fitness, identify weakness due to ageing or certain pathologies, and monitor the progress of training programmes and injury recovery [3]. Isokinetic dynamometers make it possible to assess strength in any of its manifestations (isometric, concentric and eccentric) at different speeds [4,5], and can also be used to evaluate muscle balance during a movement [6]. Moreover, isokinetic assessment makes it possible to objectively evaluate muscle performance safely, reliably and with validity [7].

Currently there are different instruments for assessing the morphological characteristics of myotendinous structures among which we can mention ultrasonography. The use of ultrasonography has become a standard method for measuring the mechanical properties of the tendon in vivo [8], like stiffness which can be assessed reliably and with validity using sonoelastography [9,10]. The majority of studies which use sonoelastography to evaluate tendon stiffness are focused on assessing the Achilles tendon [10,11], although some recent studies on the patellar tendon (PT) can also be found [12,13,14,15]. Porta et al. [13] assessed the reliability and reproducibility of sonoelastography for describing the stiffness of the patellar tendon and found that a healthy patellar tendon presents high elasticity with excellent values of intra-examiner and inter-examiner reproducibility. Some authors have related greater stiffness in the tendon with tendinopathies that involved pain and dysfunction [14]. Zhang et al. [14] compared the morphological and elastic properties of the PT in athletes with and without unilateral tendinopathy and found that in the former the tendons were thicker and showed more stiffness than healthy tendons. They also found that the stiffest tendons presented a greater intensity of pain with pressure (r = 0.62; *p* < 0.05) and greater dysfunction (r = −0.61; *p* < 0.05).

Another technique for measuring the contractile properties of more superficial muscles is tensiomyography, which is a non-invasive method designed to perform a contractility evaluation [16]. Tensiomyography (TMG) is based on the measurement of the radial displacement of the target muscle belly after receiving an external electrical stimulus. Small deformities indicate a high level of muscle tone and excess stiffness in the structures forming the muscle, while large deformities indicate lack of tone or fatigue [17]. Rodríguez-Ruiz et al. [18] found, by analysing the extensor and flexor muscles of the knee in volleyball players, that there was symmetry between the dominant and non-dominant side in the maximum radial displacement measured with TMG; however, they discovered differences when they compared the men’s group with the women’s group, mainly in the contractility response of the biceps femoris. 

There is a lack of studies that analyse the muscular characteristics using different measuring instruments to give a general integral view of the knee extensors. In the present study the aim was to combine these measurements evaluating the muscular strength of the quadriceps using an isokinetic dynamometer, stiffness and morphology of the PT using ultrasonography and the contractility characteristics of the rectus femoris using TMG. It was also aimed to observe the relations existing between the different measurements comparing the dominant to the non-dominant leg and men to women. Thus, the main objective of this study was to describe the differences between the dominant and non-dominant leg in contractility response and quadriceps strength, and the morphology and stiffness of the PT in a group of physically active men and women. We hypothesize that men will have higher values in the morphological and stiffness variables in the PT, in the contraction time of the rectus femoris and in the strength of the knee extensor muscles than women and an asymmetry will be found between the dominant and non-dominant leg in both groups.

## 2. Materials and Methods

### 2.1. Participants

Fifty physically active university students, of whom 36 were men and 14 were women, voluntarily participated in the study and their descriptive characteristics are presented in Table 1. The participants had had no type of injury in the previous 2 years, nor did they have any physical problem that would have prevented them from performing the maximum tests. The criterion of “physically active subjects” was used when they did physical activity at least two days a week (1–2 h per session), did not participate in competitive-level sport and had not followed any specific training programme in the previous three months. No participant had consumed supplements to increase muscle mass and improve strength nor did they consume more than 60 mg of caffeine per day (~1 cup of coffee) to avoid the effect of caffeine on the variables analyzed [19,20]. 

The women included in the study were always tested in the luteal phase. The menstrual cycle were monitored in each women before the start of the investigation through a mobile application (mycalendar, Period-tracker, Hong Kong, China). The onset and duration of the luteal phase was determined using: (a) period tracker application, (b) measurement of basal temperature and body mass changes and (c) assessment of urinary peak of the luteinizing hormone, following previous recommendations [21,22].

All the participants signed an informed consent form voluntarily before the start of the investigation. This study was approved by the Department of Physical Activity and Sports Sciences at the University of Castilla-La Mancha and by the Ethics Committee for Clinical Research in the area of Health in Toledo (number 62, on 10 June 2015).

### 2.2. General Procedure

Data collection took place at the Laboratory of Performance and Sports Re-adaptation at the Faculty of Sports Sciences in Toledo. All the participants were asked not to perform vigorous exercise during the 48 h prior to the assessment.

Height and mass were measured with Seca 700 scales with a stadiometer (Seca Ltd., Hamburg, Germany) with an accuracy of 100 g for mass and 1mm for height, following the recommendations of the Spanish Group for Kinanthropometry [23]. Body composition was assessed with dual emission X-ray absorptiometry (DXA, GE Healthcare, Lunar, Diegem, Belgium) with the participants lying supine [24]. The subjects were asked to kick a ball towards a goal and the leg with which they kicked was established as the dominant leg. For the evaluation of the morphology and elastic properties of the PT the subject lay supine on a stretcher, with the knee of the target leg flexed at 20° (0° corresponding to full extension of the knee) with a pillow underneath [12]. First the dominant leg was measured and then the non-dominant one following the methodology proposed by Cassel et al. [25]. The PT was scanned in the sagittal and axial planes, taking care to avoid anisotropy. The thickness and the sonoelastography of the tendon were measured at 50% of the tendon length (distance between the lower pole of the patella to the deep distal insertion in the tibia) with a GE Logic E9 ultrasound system (GE Healthcare, Waukesha, WI, USA) with an 8-12 MHz multi frequency linear probe (9L-D; GE Healthcare). Sonoelastography was performed by applying light repetitive compression with the hand-held transducer. The elastogram appeared within a rectangular region of interest (ROI) as a translucent color-coded real-time image superimposed on the B-mode image [26]. The color code indicated the strain of the tissues within the ROI, where red corresponded to soft elasticity, green and yellow indicated medium elasticity, and blue indicated hard elasticity. The B-mode image and elastogram were displayed side-by-side on the screen and the graph that appears on the screen standardized the amount and uniformity of compression. The best cine image derived from at least three compression–relaxation cycles was used for the assessment of the Elastography Index (EI) [26]. A higher value of EI Is related to a higher stiffness level. The ultrasound and sonoelastic evaluation of the tendon was carried out by the same researcher (FJ) who has ample proven experience in this type of measurements. The elastography (sonoelastrography) has showed good reproducibility (interday intra-class correlation coefficient ranging from 0.81–0.83) and validity [27].

The measurement of the contractility response of the rectus femoris of the quadriceps was performed using TMG both on the dominant and the non-dominant leg. Measurements were taken under static and relaxed conditions [28]. Participants were placed in a supine position with a supportive pad underneath the knee of the dominant leg, to maintain 60° knee flexion (0° = full knee extension) throughout the assessment. Hip angle was maintained and controlled with angles of hip flexion ranging from 33° to 40° (0° = full hip extension). The radial displacement of the muscle was measured perpendicularly to the muscle belly with a Dc-Dc Trans-Tek digital transducer (GK40, Panoptik, Ljubliana, Slovenia). The TMG assessment was performed using a pressure sensor placed on the muscle belly of the rectus femoris of the quadriceps, making sure that the sensor was placed perpendicularly to the muscle belly and with the pressure recommended by the manufacturers [29,30]. Sensor location was determined according to Delagi [31]. Both self-adhesive electrodes (5 × 5 cm) were placed symmetrically with respect to the sensor (Compex Medical SA, Ecublens, Switzerland); the positive electrode (anode) was placed proximal and the negative electrode (cathode) distal, 50–60 mm from measuring point. The measurement process was carried out following previous investigations [32]. Electrical stimulation was made with a TMG-S1 electrostimulator (Furlan Co., & Ltd., Ljubljana, Slovenia). To provoke the contraction a bipolar electric current, with increasing intensity and lasting a millisecond, was applied through two electrodes situated at the proximal and distal ends of the muscle [33]. A single 1-ms maximal monophasic electrical impulse (30 Volts with amplitude ranging from 60 to 100 mA) was used to elicit a twitch. The electric pulse amplitude started at 30 mA and was increased by 10 mA until maximal radial displacement (Dm) was reached. A 30 s resting period was applied between electrical stimuli to avoid potentiation effects [34].

TMG has demonstrated excellent test-retest reliability for contraction time (ICC = ~0.97, SEM = 0.9 ms), activation time (ICC = ~0.90, SEM = 0.9 ms) and Dm (ICC = ~0.96, SEM = 0.3 mm) [28,35].

Lastly, strength was assessed with a Biodex Multi-Joint System 3 isokinetic dynamometer (Biodex Medical Systems, New York, NY, USA) before which a warm-up was performed of 5 min on a Wattbike cycle ergometer (Wattbike Pro, Wattbike Ltd., Nottingham, UK) at an intensity of 100 W and a pace of 80–90 rpm. The participants sat on the device seat and were attached with belts as recommended by the manufacturer. The first test consisted of a maximum isometric contraction of the anterior and posterior musculature of the leg with a 90° flexion of the knee. First the dominant leg was evaluated and then the non-dominant leg. Three trials of 5 s were performed alternately for each muscle (quadriceps and hamstrings) resting 30 s between each. In each repetition the participant was verbally encouraged to apply the greatest force possible. 

After the assessment of isometric strength and a 10-min rest for the complete recovery of the participants, dynamic force was evaluated in concentric and eccentric actions of the anterior musculature of the thigh at a speed of 60°/s. The assessment protocol consisted of performing three series of three maximum concentric and eccentric repetitions. At all times, the participant had to perform a contraction of the anterior musculature of the thigh and was verbally encouraged to develop the greatest force possible in each of the contractions. Once the dominant leg had been assessed the non-dominant leg was assessed following the same protocol and after a rest of 2 min. 

### 2.3. Variables

The following anthropometric variables were recorded: age (years), height (cm), mass (kg) and fat %, as well as muscle mass (kg) in the dominant and non-dominant leg to calculate relative strength in the strength variables.

For the evaluation of the morphology and elastic properties of the PT measurements were made of its length (cm), thickness (cm) at the mid-point of the length, and stiffness index recorded in arbitrary units (UA) using sonoelastography applied at the mid-point of the length.

The following metrics were recorded to evaluate the contractility response of the rectus femoris: (1) the deformation or maximum radial displacement of the muscle belly in mm (Dm) which assesses muscle stiffness, (2) the contraction time in ms (Tc) which determines the time lag between the end of the activation time (10% of the Dm) until 90% of maximum deformation is reached, (3) the activation time in ms (Td) which represents the time that the muscle structure takes to reach 10% of total displacement, (4) the sustentation time in ms (Ts) which represents the theoretical time that the contraction is sustained and is calculated by determining the time that passes from when the initial deformation reaches 50% of its maximum value, until the deformation values during relaxation return to 50% of maximum deformation, and (5) the relaxation time in ms (Tr), which represents the time that passes from when the value of deformation falls from 90% to 50% after Dm. 

To assess isometric strength the following were recorded: absolute (N·m) and relative peak torque to the leg mass that was being measured (N·m/kg) of both the quadriceps and hamstrings. For dynamic strength at 60°/s absolute (N·m) and relative peak torque to the leg mass that was being measured (N·m/kg) and total work (W) were measured both in the concentric and eccentric movement, to assess the force exerted by the quadriceps. The ratio was also calculated between the isometric strength of knee flexion and extension, giving the Hamstring/Quadriceps (H/Q) ratio. 

### 2.4. Statistical Analysis

The following software was used: a Microsoft Excel (Microsoft Corporation, Redmond, WA, USA) spreadsheet to store the results of the measurements and SPSS v. 22.0 (SPSS Inc., Chicago, IL, USA) to perform the statistical calculations. Initially normality was confirmed with the Kolmogorov-Smirnov test and Levene’s test was performed to determine the homogeneity of variance. As all the variables showed a normal distribution and homogeneity of variance, two-way ANOVA with repeated measures (dominant vs. non-dominant leg), and independent measures (men vs. women) was used as an inferential test. A Student’s *t* test for independent measures was used to analyse the differences between men and women in the descriptive variables of the sample. The relationship between variables was analysed with the Pearson correlation coefficient. Effect sizes were calculated in all the pair-wise comparisons using the formula proposed by Glass et al. [36]. The magnitude was interpreted using the scale by Cohen [37]: an effect size lower than 0.2 was considered small, of around 0.5 was considered medium and of around 0.8 was considered large. Statistical significance was set at *p* < 0.05.

## 3. Results

As can be seen in Table 2, no significant differences were found between the dominant and non-dominant leg in the stiffness index of the PT either in the men’s or the women’s group. Values 51% higher (Confidence Interval (CI) 95%, from 0.12 to 0.92 UA, *p* = 0.013, ES = 0.44) were found in the women’s group compared to the men’s in PT stiffness in the non-dominant leg and a greater thickness of the PT in the men’s group compared to the women’s in both legs (Dominant: CI 95%, from 0.03 to 0.11 cm, *p* = 0.013, ES = 1.25; Non-dominant: CI 95%, de 0.03 a 0.11 cm, *p* = 0.013, ES =1.19). 

Regarding the contractility response significant differences were only found in Tc, where the women showed 10.1 ± 16.2% (CI 95%, de 0.15 a 5.27 ms, *p* = 0.038, ES = 0.45) higher values in the non-dominant leg than the dominant leg and the men recorded values that were 11.9% (CI 95%, de 0.18 a 7.20 ms, *p* = 0.040, ES = 0.69) higher than the women in the dominant leg (Table 3).

Higher values (*p* < 0.05) were found in the dominant leg in all the variables of isometric strength analysed, both in that generated by the quadriceps and by the hamstrings (Table 4). The men showed higher strength values (*p* < 0.05) both in the dominant and the non-dominant leg when the values were recorded in absolute terms, however, these differences disappeared when the values were normalised according to leg mass. No differences were found between the dominant and non-dominant leg in either the men or the women in the ratio between the force exerted by the quadriceps and that of the hamstrings (Table 4). 

The values obtained in the variables of concentric and eccentric strength in the quadriceps are shown in Table 5. 

Eccentric strength in the movement at 60°/s was 15.1 ± 22.7% greater in absolute peak torque (CI 95%, from 17.48 to 54.98 N·m, *p* < 0.001, ES = 0.49), 15.2 ± 22.1% greater in relative peak torque (CI 95%, from 1.62 to 6.00 N·m/kg, *p* = 0.001, ES = 0.49) and 23.7 ± 35.9% greater in total work (CI 95%, from 66.22 to 171.64 W, *p* < 0.001, ES = 0.62) in the dominant leg compared to the non-dominant leg in the men’s group. Similarly, in the women’s group eccentric strength in the movement at 60°/s was 18.8 ± 22.0% greater in absolute peak torque (CI 95%, from 7.61 a 67.74 N·m *p* = 0.015, ES = 0.64) 19.1 ± 21.2% greater in relative peak torque (CI 95%, de 2.23 a 9.25 N·m/kg, *p* = 0.002, ES = 0.64) and 21.2 ± 37.4% in total work (CI 95%, de 1.45 a 170.48 W, *p* = 0.046, ES = 0.65) in the dominant versus the non-dominant leg.

Regarding concentric strength no differences were found between the dominant and non-dominant leg. With regard to the differences between the men and women, higher values (*p* < 0.05) were observed in the men’s group in the metrics recorded in absolute terms (absolute peak torque and total work) however, no differences were found between both groups when the values were normalised by leg muscle mass (relative peak torque), both in the concentric and the eccentric movement.

The muscle mass of each leg correlated highly with the variables of isometric strength both in the quadriceps and the hamstrings with values between r = 0.75 and r = 0.83 (*p* < 0.05). Leg muscle mass also correlated significantly (*p* < 0.05) with the concentric and eccentric strength of the quadriceps but with values between r = 0.31 and r = 0.66, somewhat lower than those recorded in isometric strength. Lastly, it is worthy of note that leg muscle mass correlated (*p* < 0.05) with Tc recorded by TMG (dominant leg: r = 0.45, non-dominant leg: r = 0.46).

## 4. Discussion

The purpose of this study was to describe the differences between the dominant and non-dominant leg in the contractility response and strength in the quadriceps and in the morphology and stiffness of the PT in a group of physically active men and women. The results showed a greater thickness of the PT in the men in comparison with the women in both legs. Tc was greater in the non-dominant leg compared to the dominant leg in the women’s group. In all the isometric strength variables analysed higher values were recorded in the dominant leg as was true for eccentric strength (absolute and relative peak torque) in the movement at 60°/s in the dominant compared to the non-dominant leg in both the men’s and the women’s group. 

Regarding the thickness of the PT in our study we did not find differences between both legs in either of the groups. All the studies that have related thickness or stiffness of the tendon with pain and functional impairment have compared damaged tendons with the healthy contralateral tendon [14,38], so that it is possible that the greater thickness found in the men’s group, as it was similar in both legs, is due to their different physical constitution from that of women, as none of the subjects had any pathology or pain in their lower limbs. 

In the values for contractility response, significant differences were only found in Tc, where the women recorded a lower value in their dominant leg than in their non-dominant leg, and also than the dominant leg in the men’s group. Rodríguez-Ruiz et al. [18] found higher values for Tc in a group of men compared to women when studying volleyball players. These authors justify these differences due to the morphological and functional characteristics of the musculature, which are different in the men’s group compared to the women’s group because the shape and proportions of the pelvis, as well as the position of the bony structures of the lower limb, are especially important in this sport. Hip width and external tibia rotation prompt flexion-extension of the knee, leading to a higher probability of injury among women [18]. Higher values of Tc have also been related to muscle fatigue [17]. The Tc values in both groups and legs were very near to 30 ms, the reference value after which there is a predominance of slow twitch muscle fibres [29]. In any case, the values obtained were lower than those found by Garcia-Manso et al. [17] when analysing a group of triathletes (Tc = 63.5 ± 13.1 ms) who had a predominance of slow twitch fibres. The Dm values obtained in our study were very near to those shown by Rodríguez-Ruiz et al. [18] and no differences were seen in the rest of the TMG variables (Ta, Tr and Ts) between the dominant and non-dominant leg or between the men and the women. Like other authors, we found considerable lateral symmetry in the lower limbs of the whole sample [17,18]. The bilateral symmetry in the TMG variables found in the present study can be used by health science professionals as a preventive method to indicate if the muscle might be at risk of a future injury in the case of finding bilateral differences in any of the parameters recorded using tensiomyography. 

In all the measurements of absolute strength both in the isometric test and the isokinetic test at 60°/s the men recorded higher values than the women, however, when the values were normalised for leg muscle mass these differences disappeared in all cases. This indicates that the greater strength shown by men is due to the quantity of muscle mass and not its quality. We can underline that the age of the study subjects was between 20 and 35 years, which Murray et al. [39] consider as the moment in which the highest strength values can be attained. 

Regarding isometric strength, higher values (*p* < 0.05) were observed in the dominant side compared to the non-dominant side in both knee flexion and extension. These indicate strength asymmetries between both limbs which may be due to the greater muscular development of the preferred limb for performing activities. In our study the percentages of difference between the dominant and non-dominant side were higher in knee flexion (men: 29.9 ± 15.6% and women: 14.9 ± 19.4%) and somewhat lower in knee extension (men: 7.9 ± 16.7% and women: 15.2 ± 21.3%). It is worth underlining that these muscular asymmetries were maintained in the eccentric test at 60°/s, however, no differences were found between legs in the concentric test at 60°/s. Knapik et al. [40] established that the subjects who showed muscular imbalance between limbs of over 15% in bilateral comparison, were 2.6 times more likely to be injured compared to the subjects whose difference was lower than 15%. Other authors have also associated bilateral asymmetries with a greater risk of injury [41]. 

The ratio of strength between knee flexion and extension (hamstring/quadriceps ratio) in isometric strength was slightly lower than that recorded by other authors like Gonzalez-Rave et al. [42] (from 0.62 to 0.69) and Maly et al. [43] (from 0.58 to 0.65) who calculated it from the force exerted in extension and flexion in an isokinetic movement at 60°/s and 180°/s. This indicates that the muscular imbalance between knee agonists and antagonists, in our study, is greater than that recorded by other authors [42,43] and that the subjects should carry out compensatory exercises to increase the strength of the knee flexors, mainly the hamstrings to balance the strength between both muscle groups. 

As a novel approach, in our study we were able to relate the measurements taken with three complex technologies that are used to assess different morphology and contractility properties like isometric dynamometry, TMG and ultrasonography. In this respect, we have not found a relationship between the stiffness presented by the tendon and that presented by the muscle either in the dominant (r = 0.01; *p* = 0.937) or non-dominant (r = −0.02; *p* = 0.919) leg, which indicates that the stiffness of both structures is independent. We should bear in mind that the group that was selected to study is very homogeneous so that it is more complicated to find high correlations. Lastly, we can underline the relationship found between the muscle mass of the leg with Tc which indicates that the greater the muscle mass the longer the contraction time measured with TMG. 

There were some limitations to this study that need to be noted. The most prominent is the limited ethnic diversity. The sample consisted of Spanish young, healthy and physical active women and men. Thus, these findings are only generalizable to a similar sample. Future studies that include more ethnic/racial minority men and women are needed. Furthermore, only 28% of the sample were women, while this number (n = 14) represents enough statistical power to make cross-comparisons between groups, it is always preferable to have balanced groups. Women were tested in the luteal phase, therefore, the results of this study could not be generalized to women who are in another phase of their menstrual period. This was a cross-sectional design, and causality between muscular strength, stiffness, morphology and contractility characteristics cannot be determined. Finally, it would be interesting to analyse interactions of current findings of the PT with muscle volume of the quadriceps which could be obtained by magnetic resonance imaging of using 3D ultrasound imaging [44,45]

## 5. Conclusions

To summarize it was found that the values of isometric and eccentric strength recorded at 60°/s were higher in the dominant leg than the non-dominant leg in both groups, as well as a greater Tc in the non-dominant leg in the women. It was also revealed that the men obtained higher values than the women in the strength variables measured in absolute terms, but these differences disappeared when the data were normalised with respect to the leg muscle mass. The men showed greater thickness of the PT in both lower limbs and a higher Tc in the rectus femoris in the dominant leg in comparison to the women. 

No relationship was found between the stiffness present in the PT and that of the rectus femoris of the quadriceps or between the variables of the contractility response measured with TMG and muscle strength measured with the isometric dynamometer, which would indicate that the parameters that these instruments measure are independent. 

## Figures and Tables

**Table 1:** Descriptive characteristics of the sample.

	Men (*n* = 36)	Women (*n* = 14)
Age (years)	21.11 ± 1.88	21.64 ± 3.59
Height (cm)	174.64 ± 6.87	162.07 ± 6.88 *
Mass (Kg)	70.93 ± 10.14	58.17 ± 11.44 *
% Fat	18.47 ± 5.38	29.57 ± 7.04 *

* = Significant differences at *p* < 0.05 compared with the men’s group.

**Table 2:** Morphological and stiffness variables in the patellar tendon.

Variables	Men (*n* = 36)	Women (*n* = 14)	Main Effects of ANOVA F (*p*-Value)		
			Leg	Sex	Leg × Sex
Sonoelastography (U.A.)					
Dominant	1.18 ± 0.55	1.50 ± 0.87	0.88 (0.353)	5.13 (0.028)	1.39 (0.243)
Non-dominant	1.00 ± 0.36	1.52 ± 1.07 *			
Length (cm)						
Dominant	4.57 ± 0.50	4.35 ± 0.42	0.69 (0.411)	1.93 (0.172)	0.06 (0.812)
Non-dominant	4.52 ± 0.51	4.32 ± 0.42			
Thickness (cm)					
Dominant	0.37 ± 0.07	0.30 ± 0.04 *	0.54 (0.466)	19.08 (<0.001)	0.01 (0.996)
Non-dominant	0.38 ± 0.06	0.31 ± 0.05 *			

* = Significant differences at *p* < 0.05 compared with the men’s group.

**Table 3:** Variables of the contractility response of the rectus femoris of the quadriceps.

Variables	Men (*n* = 36)	Women (*n* = 14)	Main Effects of ANOVA [F (*p*-Value)]		
			Leg	Sex	Leg × Sex
Contraction time (ms)					
Dominant	31.09 ± 5.70	27.40 ± 5.06 *	1.71 (0.197)	1.54 (0.220)	5.31 (0.026)
Non-dominant	30.34 ± 4.92	30.12 ± 6.99 #			
Maximum displacement (mm)					
Dominant	8.83 ± 2.65	7.62 ± 2.36	0.24 (0.627)	3.09 (0.085)	0.07 (0.798)
Non-dominant	8.91 ± 1.95	7.88 ± 2.08			
Activation time (ms)					
Dominant	24.32 ± 2.28	25.33 ± 3.55	0.35 (0.555)	1.18 (0.283)	0.23 (0.634)
Non-dominant	24.87 ± 2.75	25.39 ± 2.92			
Relaxation time (ms)					
Dominant	119.10 ± 188.03	210.69 ± 286.99	0.08 (0.784)	0.75 (0.392)	1.28 (0.263)
Non-dominant	159.17 ± 202.24	144.89 ± 154.18			
Sustentation time (ms)					
Dominant	213.04 ± 196.57	290.70 ± 305.33	0.05 (0.830)	1.83 (0.182)	0.06 (0.814)
Non-dominant	237.19 ± 193.10	289.58 ± 293.48			

* = Significant differences at *p* < 0.05 compared with the men’s group. # = Significant differences at *p* < 0.05 compared with the dominant side.

**Table 4:** Variables of isometric strength of the quadriceps and hamstrings, recorded with the isokinetic dynamometer.

Variables	Men (*n* = 36)	Women (*n* = 14)	Main effects of ANOVA F (*p*-Value)		
			Leg	Sex	Leg × Sex
Absolute peak torque Quadriceps (N*m)					
Dominant	274.97 ± 68.55	181.00 ± 33.18 *	11.56 (0.001)	25.71 (<0.001)	0.35 (0.559)
Non-dominant	259.57 ± 75.20 #	159.15 ± 24.59 *#			
Absolute peak torque Hamstrings (N*m)					
Dominant	125.28 ± 30.79	78.88 ± 12.28 *	14.30 (<0.001)	27.25 (<0.001)	0.93 (0.339)
Non-dominant	110.86 ± 32.83 #	70.33 ± 15.58 *#			
Relative peak torque Quadriceps (N*m/kg)					
Dominant	28.51 ± 4.89	28.02 ± 5.32	14.07 (<0.001)	0.85 (0.361)	1.91 (0.174)
Non-dominant	26.95 ± 5.75 #	24.64 ± 4.62 #			
Relative peak torque Hamstrings (N*m/kg)					
Dominant	12.98 ± 2.35	12.17 ± 1.81	14.96 (<0.001)	1.17 (0.284)	0.04 (0.848)
Non-dominant	11.50 ± 2.79 #	10.83 ± 2.33 #			
Ratio Hamstrings/Quadriceps					
Dominant	0.46 ± 0.09	0.44 ± 0.11	0.70 (0.407)	0.023 (0.881)	0.72 (0.401)
Non-dominant	0.44 ± 0.09	0.45 ± 0.09			

* = Significant differences at *p* < 0.05 compared with the men’s group. # = Significant differences at *p* < 0.05 compared with the dominant side.

**Table 5:** Variables recorded with the isokinetic dynamometer of concentric and eccentric strength of the quadriceps.

Variables	Men (*n* = 36)	Women (*n* = 14)	Main Effects of ANOVA F (*p*-Value)		
			Leg	Sex	Leg × Sex
Absolute concentric peak torque at 60°/s (N*m)					
Dominant	178.63 ± 45.64	137.45 ± 24.44 *	0.37 (0.545)	18.11 (<0.001)	0.90 (0.347)
Non-dominant	180.41 ± 39.26	129.28 ± 21.18 *			
Relative concentric peak torque at 60°/s N*m/kg)					
Dominant	18.93 ± 5.26	21.26 ± 3.82	1.23 (0.274)	1.79 (0.187)	1.30 (0.260)
Non-dominant	18.95 ± 3.83	19.95 ± 3.38			
Total concentric work at 60°/s (W)					
Dominant	327.43 ± 121.37	234.20 ± 69.74 *	1.24 (0.271)	9.27 (0.004)	0.01 (0.926)
Non-dominant	340.28 ± 108.23	249.40 ± 61.77 *			
Absolute eccentric peak torque at 60°/s (N*m)					
Dominant	263.19 ± 64.49	184.31 ± 60.80 *	17.59 (<0.001)	15.16 (<0.001)	0.01 (0.935)
Non-dominant	226.96 ± 83.29 #	146.64 ± 57.63 *#			
Relative eccentric peak torque at 60°/s (N*m/kg)					
Dominant	27.64 ± 7.01	28.29 ± 8.73	21.57 (<0.001)	0.02 (0.896)	0.88 (0.353)
Non-dominant	23.82 ± 8.58 #	22.55 ± 9.09 #			
Total eccentric work at 60°/s (W)					
Dominant	511.19 ± 185.51	305.29 ± 141.46 *	17.11 (<0.001)	14.14 (<0.001)	0.44 (0.509)
Non-dominant	392.26 ± 199.09 #	219.33 ± 123.40 *#			

* = Significant differences at *p* < 0.05 compared with the men’s group. # = Significant differences at *p* < 0.05 compared with the dominant side.

## Data Availability

The data presented in this study are available on request from the corresponding author. The data are not publicly available due to restrictions of the subjects’ agreement.
